# Effect of Black Tea Extract and Thearubigins on Osteoporosis in Rats and Osteoclast Formation *in vitro*

**DOI:** 10.3389/fphys.2018.01225

**Published:** 2018-09-03

**Authors:** Qingqing Liang, Ming Lv, Xiaojuan Zhang, Jun Hu, Ying Wu, Yewei Huang, Xuanjun Wang, Jun Sheng

**Affiliations:** ^1^Key Laboratory of Pu-er Tea Science, Ministry of Education, Yunnan Agricultural University, Kunming, China; ^2^Tea Research Center of Yunnan, Kunming, China; ^3^College of Food Science and Technology, Yunnan Agricultural University, Kunming, China; ^4^College of Science, Yunnan Agricultural University, Kunming, China; ^5^State Key Laboratory for Conservation and Utilization of Bio-Resources in Yunnan, Kunming, China

**Keywords:** osteoporosis, black tea extract, thearubigins, osteoclast, osteoclastogenesis

## Abstract

**Background:** Osteoporosis is a major health problem that is closely related to substantial morbidity, mortality and decline in life quality for the aging population. Although previous studies and epidemiological evidence have demonstrated an association between black tea consumption and the prevention of bone loss, the underlying mechanism remains unclear. So, the effect of black tea extract (BTE) and thearubigins (TRs) on osteoporosis in rats and osteoclast formation *in vitro* were investigated.

**Methods:** *In vivo*, ovariectomized (OVX) rats were used to establish osteoporosis models. To validate the model and study the effects of BTE and TRs on osteoporosis, the female Wistar rats were divided into a sham-operated group and five OVX groups including model, Xian-Ling-Gu-Bao (XLGB) (as a positive control), BTE, TRs low-dose, and TRs high-dose group. The rats in the four treatment groups were given the corresponding test sample for 12 weeks. Then, the body weight, femur indices, and serum biomarkers were examined and analyzed. *In vitro*, RAW264.7 murine macrophages were used as model of osteoclast formation. The effects of BTE and TRs on osteoclasts formation and the specific genes and protein levels of osteoclasts were determined.

**Results:** Although there was no significant effect on the OVX-induced body weight gain by BTE or TRs, the levels of maximum bending force, cortical bone thickness and biomarker of bone resorption (acid phosphatase) can be significantly ameliorated by BTE or TRs in OVX rats. Furthermore, both of BTE and TRs can inhibit the osteoclastogenesis and diminish the expression levels of the related genes and proteins.

## Introduction

Osteoporosis is a major health problem affecting the elderly (Shen et al., 2009b) with an increasing prevalence worldwide owing to the aging population, and its diagnosis and treatment remain challenging (Maeda and Lazaretti-Castro, 2014). Osteoporosis is a degenerative disease of bone microstructure that is characterized by decreased bone mass and bone quality, which can lead to increased bone fragility and a higher risk of fracture (Shen et al., 2009b; Rachner et al., 2011; Sacco et al., 2013), and is associated with substantial morbidity, mortality, and decline in quality of life (Maeda and Lazaretti-Castro, 2014). Previous studies have shown that the incidence of osteoporosis is higher in women than in men (Shen et al., 2009a; Rakel et al., 2011), and in women with osteoporosis, adverse cardiovascular events are more common (Tasic et al., 2015).

The main goal of osteoporosis treatment is to prevent fracture, maintain or increase bone mineral density (BMD), and improve the physiological function (Tella and Gallagher, 2014). Fracture prevention is one of the public health priorities worldwide (Gambacciani and Levancini, 2014) and the current methods include both non-pharmacological management and pharmacological interventions (Tella and Gallagher, 2014). Non-pharmacological management methods include improved nutrition, calcium and vitamin D supplements, and exercise (Hosam, 2006; Tella and Gallagher, 2014). Pharmacological interventions can be classified into antiresorptive agents that prevent bone resorption and anabolic agents that help in new bone formation. The approved antiresorptive agents in the United States include hormone replacement therapy (HRT), bisphosphonates, selective estrogen receptor modulators (SERMS), and the monoclonal antibody denosumab. Anabolic agents include strontium ranelate (only available in Europe) and teriparatide (Tsai et al., 2013; Zhang et al., 2015). However, these pharmacological interventions are generally complicated by undesirable side effects such as hypercalcemia, joint pain, gastrointestinal intolerance, urinary or respiratory tract infections, increased risk of malignancy, and excessive suppression of bone turnover (Bolland et al., 2010, 2011; Tella and Gallagher, 2014). Consequently, it is necessary to find a new strategies for the prevention and treatment of osteoporosis with far fewer side effects.

Epidemiological evidence has revealed an association between tea consumption and the prevention of bone loss in the elderly population. Tea drinking can reduce the risk of osteoporotic fractures (Hegarty et al., 2000; Devine et al., 2007; Shen et al., 2011; Byun et al., 2014; Huang and Tang, 2016). Both of Green tea, black tea and Puer extracts had anti-osteoporosis effects in ovariectomized (OVX) rats or in cell study (Das et al., 2004; Vester et al., 2014; Liu et al., 2017). The beneficial effects of tea consumption on osteoporosis may be attributable to its antioxidant capability, and this capability is assumed to be mediated by the polyphenols present (Shen et al., 2009a). Black tea being one of the world’s most popular beverages (Nie et al., 2014) and during its processing the antioxidant components (catechins) are oxidized and transformed into oxidation products including thearubigins (TRs) and theaflavins (TFs). Caffeine is stable during the processing of black tea. Consequently, the main functional components of black tea are TRs, TFs, and caffeine. The active constituent TFs may effectively diminished osteoclast formation and differentiation (Oka et al., 2012). Caffeine may reduce BMD in growing rats through promoting osteoclastogenesis (Zhou et al., 2010; Liu et al., 2011). However, despite the fact that TRs are one of the most abundant constituents of black tea extract (BTE), their contribution to the ameliorative effects of BTE on osteoporosis has seldom been reported.

In this study, OVX rats and RAW264.7 murine macrophages were used to investigated the effect of BTE and TRs on osteoporosis *in vivo* and osteoclast formation *in vitro*.

## Materials and Methods

### Reagents

BTE (Black tea was boiled in water at w/v of 1/10 for 30 min three times. The decoction was then collected, concentrated, and spray-dried) and TRs were provided by the Tea Research Center of Yunnan. Xian-Ling-Gu-Bao (XLGB) capsules were bought from Guizhou Tongjitang Pharmaceutical (Guizhou, China). Recombinant mouse receptor activator of nuclear factor-κB ligand (RANKL) expressed in *Escherichia coli* was obtained from R&D Systems (Minneapolis, MN, United States) and dissolve according to instructions. 3-(4,5-Dimethylthiazol-2-yl)-2,5-diphenyltetrazoliumbromide (MTT) was obtained from Sigma Aldrich (St. Louis, MO, United States). Tartrate-resistant acid phosphatase (TRAP) staining kit was purchased from Sigma Aldrich (St. Louis, MO, United States). Anti-nuclear factor of activated T-cells cytoplasmic 1 (NFATc-1), anti-cathepsin K, and anti-c-Src antibodies were purchased from Santa Cruz Biotechnology (Santa Cruz, CA, United States). Anti-β-tubulin was purchased from Proteintech Group, Inc. (Rosemont, IL, United States) and horseradish peroxidase conjugated secondary was purchased from Thermo Fisher Scientific (Waltham, MA, United States). Chloral hydrate was purchased from China National Pharmaceutical Group Chemical Reagent Co., Ltd. The acid phosphatase (ACP) assay kit was purchased from Nanjing Jiancheng Bioengineering Institute (Nanjing, China). The bone gla protein (BGP) radioimmunoassay kit was obtained from the Beijing North Biotechnology Technology Institute (Beijing, China).

### Animals and Treatment

The animal experiments used healthy female Wistar rats provided by the Experimental Animal Center of Jilin University [12 weeks old, Cat No. SYXK-(Ji) 2010-0006]. The rats were maintained in a controlled environment (12-h light/12-h dark cycle; humidity 50–60%; ambient temperature 24 ± 1°C) and were administered standard laboratory food and water *ad libitum*. All of the animal experiments were performed in an animal facility according to institutional guidelines and were approved by the Institutional Animal Care and Use Committee of Jilin Academy of Traditional Chinese Medicine. Adverse events were not observed. After an acclimation period of 1 week, the animals were anesthetized using 6% chloral hydrate (0.5 mL/100 g) and subjected to either sham operation or surgical removal of both ovaries under sterile conditions to establish the experimental rat model of osteoporosis. After 2 weeks, the OVX rats were divided into five groups: model group, XLGB group, BTE group, TRs low-dose group, and TRs high-dose group. The animals in the XLGB group were intragastrically administered XLGB (240 mg/kg body weight), BTE group were intragastrically administered BTE (100 mg/kg body weight), while those in the low- and high-dose TRs groups were intragastrically administered TRs at dosages of 9.6 mg/kg body weight and 19.2 mg/kg body weight, respectively. The BTE and TRs dose was selected based on the LD50 of rats (1860 mg/kg and 1200 mg/kg, respectively) and pharmacologic dose (Mehri et al., 2008; Zhu et al., 2017). The model group and the sham operation group were intragastrically administered equal volumes (0.15 mL/10 g body weight) of distilled water. All of the treatments were administered once a day at 10 a.m. for three consecutive months. After 3 months, the rats were feed-deprived for 8 h before the animals were euthanized by deep ether anesthesia. Blood was obtained from the abdominal aorta and the left/right femurs were collected for subsequent study.

### Measurements of Bone Mineral Density, Strength, and Physical Quantity

The left femur was determined and the muscle tissue was removed. The femur wet weight coefficient was calculated as wet weight of the femur/body weight. The left femur was used dual-energy x-ray absorptiometry (DEXA) scans were performed to obtain BMD according to the manufacturer’s instructions (HOLOGIC discovery WA, United States). The biomechanical parameters such as fracture deflection and the maximum bending force of the left femur were measured on a CSS-44100 biomechanical tester (Changchun Research Institute for Mechanical Science Co., Ltd, China) according to the manufacturer’s instructions. The experimental conditions were stride distance 20 mm and loading speed 5 mm/s. The right femur was fixed with 10% formalin, followed by paraffin embedding treatment and tissues were cut into 4-mm sections, routine stained with hematoxylin and eosin (H&E). The BI-2000 image analysis system (Chengdu Technology and Market Co., Ltd, Chengdu, China) was used to acquired static images of the cortical structure, and the thickness of the cortical bone was measured by computer aided software (Chunxiao et al., 2016).

### Determination of Serum Biochemical Indices

The blood samples were centrifuged at 6000 rpm/min for 10 min at 4°C and the serum was collected. ACP and BGP were quantitated using the corresponding kits according to the manufacturer’s instructions.

### Cell Culture

This study used RAW264.7 murine macrophages (ATCC, Manassas, VA, United States). These cells were cultured in Dulbecco’s modified Eagle’s medium (DMEM/High Glucose, Thermo Fisher Scientific) supplemented with 4 mmol/L L-glutamine (supplier) and 10% fetal bovine serum (FBS, Biological Industries Israel Beit Haemek Ltd.). The cells were maintained at 37°C in a humidified incubator with 5% CO_2_.

### Tartrate-Resistant Acid Phosphatase (TRAP) Staining

RAW264.7 cells (3 × 10^3^ per well) were seeded in 96-well plates overnight and then incubated with the desired concentrations of XLGB, BTE, or TRs in the presence of RANKL (50 ng/mL) at 37°C for 6 days. Subsequently, the cells were stained for TRAP activity according to the instructions provided by the manufacturer and observed using a light microscope. TRAP-positive multinucleated cells containing five or more nuclei were counted as osteoclasts (Xu et al., 2018).

### Cell Viability Assay

RAW264.7 cells (3 × 10^4^ per well) were seeded in 96-well plates overnight and then incubated with the desired concentrations of BTE or TRs at 37°C for 48 h. Next, MTT (20 μL) was added to each sample and the plates were incubated for 4 h. Dimethyl sulfoxide (150 μL) was then added to each well and the samples were shaken. The absorbance was measured at 492 nm using a FlexStation3 MultiMode Microplate Reader (Molecular Devices). The cell viability was compared with that of the control group in the absence of BTE or TRs.

### Quantitative Real-Time PCR (qRT-PCR) Analysis

RAW264.7 cells (1.2 × 10^5^ per well) were seeded in 12-well plates overnight and then incubated with the desired concentrations of XLGB, BTE, or TRs in the presence of RANKL (50 ng/mL) at 37°C for 48 h. The total RNA was isolated from the cell cultures using TransZol Up (TransGen Biotech, Beijing, China) according to the manufacturer’s protocol. The first-strand cDNA was created using a PrimeScript RT Reagent Kit with gDNA Eraser (TaKaRa Bio, Otsu, Japan). qRT-PCR was performed using SYBR Premix Ex Taq^TM^ II (Tli RNaseH Plus, TaKaRa Bio) following the manufacturer’s protocol. The oligonucleotide primers were designed by Shanghai Generay Bioengineering Co., Ltd., Glyceraldehyde-3-phosphate dehydrogenase (GAPDH) mRNA was used as an internal reference for the expression of mRNA. The final experimental results were obtained using a 7900HT Fast Real-Time PCR system (Applied Biosystems, Foster City, CA, United States). All data were analyzed using the 2^−ΔΔCT^ method and are expressed as ratios relative to GADPH (Liu et al., 2017). The primer sequences are listed in **Table 1**.

**Table 1:** Primers used in the qRT-PCR study.

Gene	Forward (5′–3′)	Reverse (5′–3′)
*GADPH*	AACTTTGGCATTGTGGAAGG	ACACATTGGGGGTAGGAACA
*TRAP*	GCTGGAAACCATGATCACCT	GAGTTGCCACACAGCATCAC
*NFATc-1*	TGGAGAAGCAGAGCACAGAC	GCGGAAAGGTGGTATCTCAA
*Cathepsin K*	CTTCCAATACGTGCAGCAGA	TCTTCAGGGCTTTCTCGTTC
*c-Src*	CCAGGCTGAGGAGTGGTACT	CAGCTTGCGGATCTTGTAGT

### Immunoblotting Analysis

RAW264.7 cells (4 × 10^5^ per well) were seeded in 60-mm plates overnight and then incubated with the desired concentrations of XLGB, BTE, or TRs in the presence of RANKL (50 ng/mL) at 37°C for 48 h. Cell lysis was performed using RIPA buffer (Solarbio, Beijing, China) according to the manufacturer’s protocol. The proteins were separated using 10% SDS-PAGE and transferred onto a PVDF membranes (EMD Millipore Corporation, Merck Life Sciences KGaA, Darmstadt, Germany) (Niu et al., 2017). The membranes were then incubated at 4°C overnight with the primary antibodies anti-NFATc-1, anti-cathepsin K, anti-c-Src, and anti-β-tubulin, followed by the appropriate combination of secondary antibodies according to the manufacturer’s protocol. The images were obtained using a FluorChem E system (ProteinSimple, Santa Clara, CA, United States).

### Statistical Analysis

All data were analyzed using SPSS 17.0 (IBM Corp., Chicago, IL, United States) and Graph Pad Prism 5 (GraphPad Software, Inc., La Jolla, CA, United States) for Windows. All values in the text are expressed as mean ± standard error of the mean (SEM). The “Linear Mixed Models” was used to analyze the data of body weight. For other data related to bone metabolism, “Independent Samples *T*-Test” and “One way ANOVA” were used for the comparison of sham vs model group and model vs treatment groups using pooled variance, respectively. A probability of *p* < 0.05 was considered significant.

## Results

### Principal Components Analysis of BTE

The content of TRs was determined by spectrophotometer (Roberts and Smith, 1963; Owuor and Obanda, 1995), caffeine and three kinds of TFs (TF, TF-3′, and TF-3,3′) were determined by high performance liquid chromatograph (HPLC) (Wang et al., 2004; Zhu et al., 2017) (**Supplementary Figures 1**, **2**). The results showed that TRs, caffeine, and title three kinds of TFs, account for 6.72%, 39.6%, 0.07%, respectively.

### Effect of BTE and TRs on the Body Weight of OVX Rats

**FIGURE 1:**
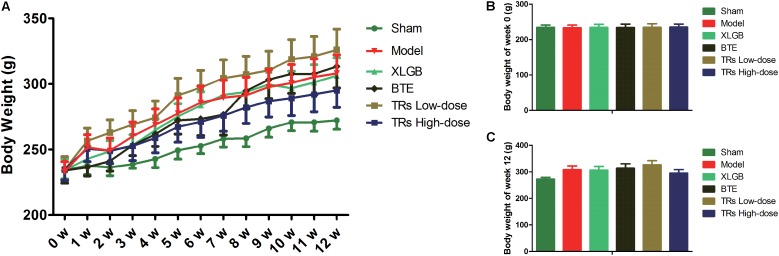
Effects of black tea extract (BTE) and thearubigins (TRs) on body weight (g) in OVX rats. **(A)** Growth curve for each group, **(B)** the initial (week 0) body weight, **(C)** the final (week 12) body weight. The initial, weekly, and final weights of the rats were measured throughout the experiment and the data were recorded once a week. All data are presented as mean ± SEM (*n* = 12). The data was analyzed using “Linear Mixed Models.” The model group *vs* sham group and each treatment group *vs* model group were analyzed, respectively.

To verify the role of BTE and TRs in ameliorating osteoporosis, an experimental model of osteoporosis was established in female rats by the surgical removal of both ovaries. To investigate the effect of BTE and TRs on the body weight of OVX rats, the body weight were recorded once a week (**Figure 1**). The results showed that the body weight of the rats in each group increased gradually during the 12 weeks (*p* < 0.05). Compared with sham group, the body weight gain induced by ovariectomy was observed (*p* = 0.048). However, there was no significant difference in body weight between model group and each treatment group (*p* > 0.05). These results indicate that the OVX-induced body weight gain can not be down-regulated by the treatment with XLGB, BTE, or TRs.

### Effect of BTE and TRs on Femoral Physical Parameters and Bone Quality in OVX Rats

**FIGURE 2:**
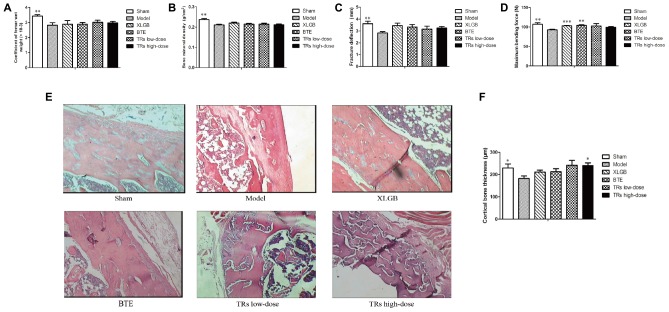
The effect of black tea extract (BTE) and thearubigins (TRs) on bone quality in ovariectomized (OVX) rats. **(A)** Femur wet weight coefficient, **(B)** bone mineral density, **(C)** femoral fracture deflection, **(D)** maximum bending force for each treatment group. **(E)** The cortical bone tissue was examined by hematoxylin and eosin (H&E) staining (magnification × 400) and **(F)** cortical bone thickness counts, statistical analysis. All data are presented as mean ± SEM (*n* = 10). “Independent Samples T-Test” and “One way ANOVA” were used for the comparison of sham vs model group and model vs treatment groups using pooled variance, respectively. ^∗^*p* < 0.05, ^∗∗^*p* < 0.01, and ^∗∗∗^*p* < 0.001 *vs* model group.

We next studied the femur indices. The results showed that the femur wet weight coefficient, BMD, femoral fracture deflection, maximum bending force and cortical bone thickness of the model group were lower than those of the sham group (*p* < 0.05; **Figure 2**). Compared with the model group, the level of maximum bending force is significantly higher in XLGB and BTE group (**Figure 2D**). For the level of the cortical bone thickness, it’s significantly higher in TRs high-dose group than model group (**Figures 2E,F**). Nevertheless, the effects of BTE and TRs on the femur wet weight coefficient, BMD and femoral fracture deflection were not significant (**Figures 2A–C**). These results suggest that the OVX-induced negative effects on the femur indices was significant, and some indicators can be significantly improved by treatment with BTE or TRs and the effect is similar to XLGB.

### The Serum Biochemical Parameters Can Be Ameliorated by BTE and TRs in OVX Rats

**FIGURE 3:**
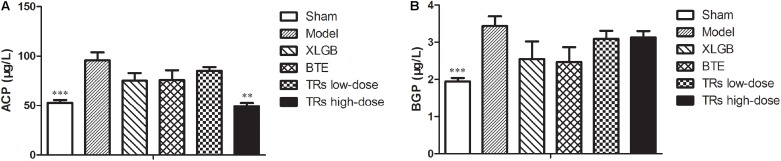
The effect of black tea extract (BTE) and thearubigins (TRs) on the serum biochemical indices in ovariectomized (OVX) rats. The levels of **(A)** acid phosphatase (ACP) and **(B)** bone gla protein (BGP) for the different treatment groups. All data are presented as mean ± SEM (*n* = 10). “Independent Samples T-Test” and “One way ANOVA” were used for the comparison of sham vs model group and model vs treatment groups using pooled variance, respectively. ^∗∗^*p* < 0.01 and ^∗∗∗^*p* < 0.001 *vs* model group.

Then, to further elucidate the effects of BTE and TRs on osteoporosis, the serum biomarkers of bone resorption (ACP) and bone formation (BGP) were determined. Compared with the sham group, the levels of ACP and BGP were significantly higher than model group. On the other hand, high-dose TRs treatment could significantly lower the high level of ACP induced by OVX (**Figure 3A**). However, although BGP levels in the four treatment groups appear to be lower than model group, there was no significant difference was observed (**Figure 3B**). These results demonstrate that the OVX-induced negative effects on serum biochemical parameters was significant, and the ACP level can be significantly ameliorated by high-dose TRs in OVX rats.

### Osteoclast Formation Can Be Inhibited by BTE

RAW264.7 murine macrophages have been proved to be used as a model for the formation of osteoclasts induced by RANKL *in vitro* (Wang et al., 2015; Niu et al., 2017). So, we further investigated the effect of BTE and TRs on RANKL-induced osteoclastogenesis in RAW264.7.

**FIGURE 4:**
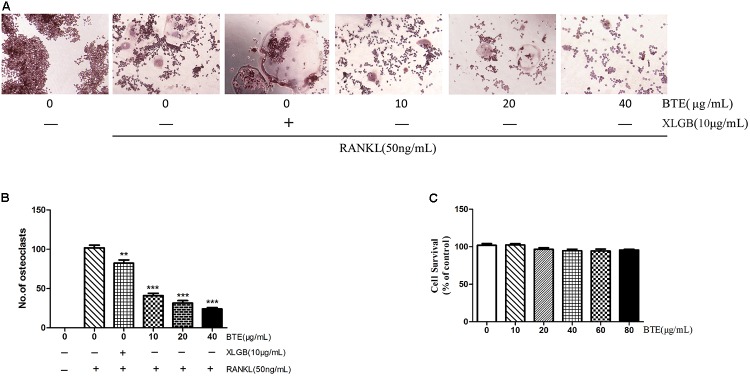
The effect of black tea extract (BTE) on osteoclastogenesis. **(A)** The role of BTE in the differentiation of RAW264.7 cells into osteoclasts was determined by tartrate-resistant acid phosphatase (TRAP) staining (magnification × 200). **(B)** The TRAP staining results were analyzed, where TRAP-positive multinucleated cells containing five or more nuclei were counted as osteoclasts. **(C)** The survival of RAW264.7 cells in the presence of various concentrations of BTE was determined using the MTT assay. All data are presented as mean ± SEM (*n* = 3). “One way ANOVA” was used for the comparison of BTE treatment *vs* only RANKL treatment. ^∗∗^*p* < 0.01and ^∗∗∗^*p* < 0.001 *vs* only RANKL treatment.

Initially, we investigated the effects of BTE on the osteoclastogenesis induced by RANKL. First, the effects of BTE (10, 20, and 40 μg/mL) on the osteoclast formation were investigated by TRAP staining. The results demonstrated that the osteoclast formation can be inhibited by BTE in a concentration-dependent manner (*p* < 0.01; **Figures 4A,B**). Then, to evaluate the cytotoxicity of BTE, the effects of different concentrations (10, 20, 40, 60, and 80 μg/mL) of BTE on the survival rate of RAW264.7 cells were examined. The results revealed that no obvious effects on cell viability within the range of BTE concentrations tested (*p* > 0.05, **Figure 4C**).

### RANKL-Induced Osteoclast-Specific Genes and Proteins Expression Were Diminished by BTE

**FIGURE 5:**
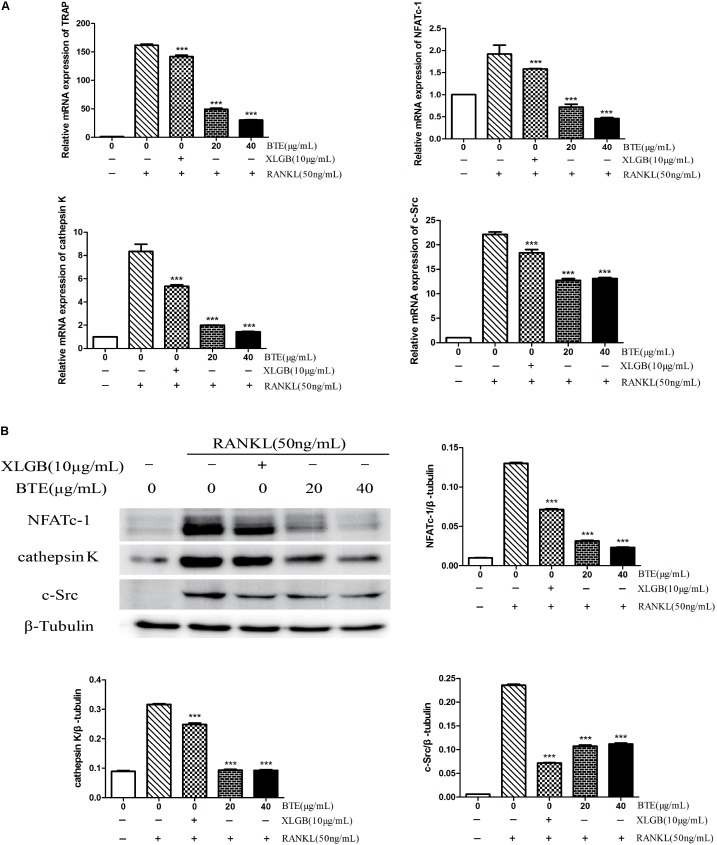
The Effects of black tea extract (BTE) on the expression of genes and proteins related osteoclastogenesis. The levels of specific genes and proteins were determined using qRT-PCR and western blotting for osteoclasts treated with BTE. **(A)** The mRNA expression levels of TRAP, NFATc-1, cathepsin K, and c-Src. **(B)** The protein expression levels of NFATc-1, cathepsin K, and c-Src and the results were quantified using AlphaView software. All data are presented as the mean ± SEM (*n* = 3). “One way ANOVA” was used for the comparison of BTE treatment *vs* only RANKL treatment. ^∗∗∗^*p* < 0.001 *vs* only RANKL treatment.

To further explore the inhibitory effect of BTE on osteoclasts, the effects of BTE on the levels of genes associated with osteoclastogenesis were examined. The mRNA levels of TRAP, NFATc-1, cathepsin K, and c-Src were decreased by XLGB and BTE (*p* < 0.001; **Figure 5A**). Similarly, the expression levels of the corresponding proteins, NFATc-1, cathepsin K, and c-Src, were also significantly inhibited by XLGB and BTE (**Figure 5B**). Taken together, these results indicate that osteoclast formation can be inhibited by BTE at the cellular and molecular levels.

### TRs Could Be One of the Main Functional Ingredient of BTE for Inhibition of Osteoclastogenesis

**FIGURE 6:**
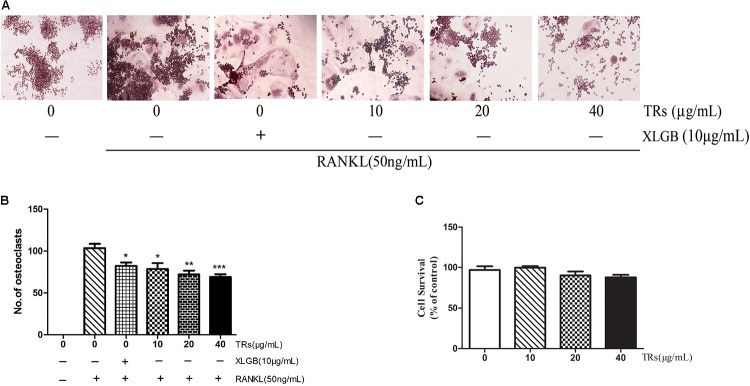
The effect of thearubigins (TRs) on osteoclastogenesis. **(A,B)** The TRAP staining test was used to determine the effects of TRs on the differentiation of RAW264.7 cells into osteoclasts (magnification × 200). The numbers of osteoclasts in each sample were counted and quantified. **(C)** The MTT assay was used to determine the survival of RAW264.7 cells in the presence of the different concentrations of TRs. All data are presented as the mean ± SEM (*n* = 3). “One way ANOVA” was used for the comparison of BTE treatment *vs* only RANKL treatment. ^∗^*p* < 0.05, ^∗∗^*p* < 0.01, and ^∗∗∗^*p* < 0.001 *vs* only RANKL treatment.

TFs have been reported to inhibit osteoclastogenesis (Oka et al., 2012; Nishikawa et al., 2015). The structure of some TRs are similar to those of TFs and TRs can be formed by the further oxidation and polymerization of TFs (Menet et al., 2004). The results of composition analysis showed that the content of TRs in BTE is about 40%. Nevertheless, the content of TFs in our BTE sample is little (less than 0.1%). Accordingly, in order to test the hypothesis that TRs could be one of the main functional component of BTE for the effect on the suppression of osteoclast formation, the effects of TRs on the RANKL-induced differentiation from RAW264.7 cells into osteoclasts were also investigated by TRAP staining. The results were similar to those observed for BTE, in that RANKL-induced osteoclastogenesis was inhibited by TRs, which TRs in a concentration-dependent manner in the range from 10 to 40 μg/mL (*p* < 0.05, *p* < 0.01, or *p* < 0.001; **Figures 6A,B**). Then, the cytotoxicity of TRs was also evaluated using the MTT assay. As expected, the results showed that TRs has no obvious effects on cell viability up to a concentration of 40 μg/mL (*p* > 0.05, **Figure 6C**).

### RANKL-Induced Osteoclast-Specific Genes and Proteins Expression Were Also Ameliorated by TRs

**FIGURE 7:**
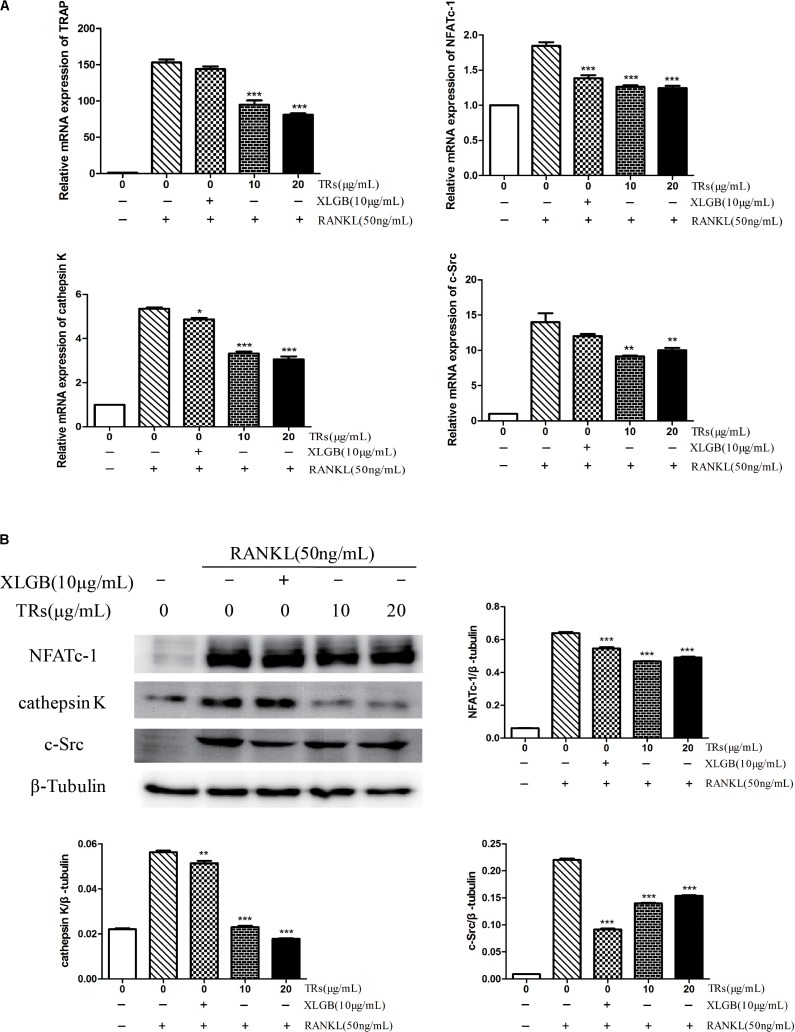
The Effects of thearubigins (TRs) on the expression of genes and proteins related osteoclastogenesis. Thearubigins (TRs) reduce the levels of genes and proteins related to osteoclast formation. The levels of specific genes and proteins were determined by qRT-PCR and western blotting for osteoclasts treated with TRs. **(A)** The mRNA expression levels of TRAP, NFATc-1, cathepsin K, and c-Src. **(B)** The protein expression levels of NFATc-1, cathepsin K, and c-Src and the results were quantified using AlphaView software. All data are presented as the mean ± SEM (*n* = 3). “One way ANOVA” was used for the comparison of BTE treatment *vs* only RANKL treatment. ^∗^*p* < 0.05, ^∗∗^*p* < 0.01, and ^∗∗∗^*p* < 0.001 *vs* only RANKL treatment.

The mRNA levels of TRAP, NFATc-1, cathepsin K, and c-Src were found to be ameliorated by TRs (*p* < 0.05, **Figure 7A**). Similarly, the expression levels of the corresponding proteins, NFATc-1, cathepsin K, and c-Src, were also inhibited by TRs (*p* < 0.01, **Figure 7B**). It is noteworthy that the concentration of TRs used was half that of BTE, and the effect is similar to XLGB.

## Discussion

Almost two-thirds of the world’s population consume tea every day and black tea comprises approximately 80% of all tea consumed (Huang et al., 2009; Nie et al., 2014; Lin et al., 2017). An epidemiological studies suggested that postmenopausal women who drank tea had higher BMD measurements (Hegarty et al., 2000; Nash and Ward, 2016). As we all known, TRs and TFs are the major functional constituents found in black tea and are responsible for its unique taste and bright red/orange color. Furthermore, TRs can be formed from TFs by further oxidation, have a higher molecular weight, and are poorly characterized both chemically and biochemically. And black tea has been well documented to have health-promoting effects because of the TRs and TFs (Das et al., 2005).

The bone loss in models of osteoporosis was reported to be abrogated by epigallocatechin-3-agallate or TFs, which revealed the role of tea polyphenols and their oxidation products in variety of bone disorders (Oka et al., 2012; Nishikawa et al., 2015). On the contrary, caffeine may reduce BMD in growing rats through promoting osteoclastogenesis (Zhou et al., 2010; Liu et al., 2011). In addition, polysaccharide could also be responsible for the inhibition effect of osteoclast formation (Xu et al., 2018). The results of composition analysis showed that the content of TRs in BTE is about 40%. Nevertheless, the content of TFs in our BTE sample is little (less than 0.1%). As analogs of TFs that have undergone more extensive oxidation, the content of TRs is much higher than that of TFs (Fukuda et al., 2005; Samanta et al., 2015), we speculate that TRs also has good preventive effect on osteoporosis.

With the rapidly aging society, osteoporosis has become a social question that needs to be solved (Lkhagvasuren et al., 2015; Miyamoto, 2015). To date, although the anti-osteoporotic effects of BTE have been demonstrated in OVX rats (Das et al., 2005, 2013), but the mechanism remained unclear. Then, to investigate the effects of BTE and TRs on ameliorating osteoporosis *in vivo*, the effects of BTE and TRs on experimental measures of bone metabolism were examined in OVX rats. The animal experiment results revealed that the maximum bending force, cortical bone thickness and ACP can be improved by administering BTE or TRs in OVX rats. The high-dose TRs had a tendency to down-regulate the OVX-induced body weight gain from the fourth week after ovariectomy, although there was no significant difference.

Corresponding to osteoclast formation, level of ACP can also be used as a indicator of bone resorption (Liu et al., 2017). Our result showed that ACP level was enhanced in OVX rats could be markedly attenuated by high-dose TRs. Contrary to ACP, the level of BGP was widely applied as a marker of bone formation (Ye et al., 2015). The result showed that BGP level was enhanced in OVX rats (*p* < 0.001). Nevertheless, the high level of BGP induced by OVX could not be markedly attenuated by TRs. These results indicate that the mechanism of the effect of TRs on osteoporosis may be mainly through inhibition of bone absorption rather than of bone formation.

Previous studies have indicated that RANKL, as one of the major cytokines released by osteoblasts, plays a very important role in the initiation of osteoclast differentiation and their bone resorptive function (Chen et al., 2017). RANKL-induced RAW264.7 cell line was widely used to establish osteoclast formation model (Shui et al., 2002; Hirotani et al., 2004; Baek et al., 2016; Liu et al., 2017). Consequently, in our study, RAW264.7 murine macrophages induced by RANKL were used to research the effect of BTE and TRs in ameliorating osteoporosis at the cellular level. The results indicate that RANKL-induced osteoclast formation can be inhibited by BTE and TRs at the cellular and molecular levels. Interestingly, compared with BTE, relatively lower dose of TRs has a similar effect. Therefore, we consider TRs could be one of the main functional component of BTE for inhibiting osteoclast formation.

Xian-Ling-Gu-Bao is a well-known traditional Chinese patent medicine, widely used in the treatment of osteoporosis, osteoarthritis, aseptic bone necrosis, and bone fracture (Cheng et al., 2013; Gao et al., 2013). The Chinese State Food and Drug Administration has officially approved it for use in Traditional Chinese Medicine (Wu et al., 2017). In the animal experiments, similar with XLGB treatment, BTE and TRs treatment could also ameliorated the level of femoral index and serum biochemical markers, and the effects of BTE and TRs were similar to XLGB. For the cell experiment, BTE and TRs have relatively better effects on the inhibition of osteoclast formation than XLGB. These results suggest that BTE and TRs have similar effects on osteoporosis with XLGB.

The results showed that the effect of TRs is better than that of BTE in animal experiment. Curiously, the effect of TRs is not as good as that of BTE in cell experiments. Firstly, inhibition of osteoclast differentiation may be only one of the mechanisms of the anti-osteoporosis effect for TRs. There may be other pathways for TRs to alleviate osteoporosis. Other possible mechanisms were investigated in previous studies. For instance, ovariectomy-induced bone decay can be diminished by BTE through increasing the serum estradiol levels (Das et al., 2005). In addition, bone loss in models of osteoporosis can be abrogated by theaflavin-3,3′-digallate via a mechanism possibly involving the inhibition of DNA methyltransferase (Nishikawa et al., 2015). The question of whether the functions of estradiol regulation and DNA methyltransferase inhibition are the main mechanisms underlying the effects of BTE and TRs on osteoclastogenesis deserves further investigation. Secondly, osteoclast formation could also be inhibited by other functional components, for example by gallic acid, saponin, and so on.

To the best of our knowledge, this study has demonstrated for the first time that osteoclast formation can be inhibited by BTE and TRs at the cellular level and that TRs could be one of the functional component of BTE, as determined both *in vivo* and *in vitro*. Recent research into the effects of Puer tea on osteoporosis obtained similar results, demonstrating that Puer tea can ameliorate ovariectomy-induced osteoporosis in rats and suppress osteoclastogenesis *in vitro* (Liu et al., 2017). Further research is needed to identify the main functional components of BTE and elucidate the action mechanism of TRs on ameliorattion of osteoporosis.

## Conclusion

In conclusion, the present study demonstrates that TRs could be one of the functional component of BTE for ameliorating ovariectomy-induced osteoporosis in rats and inhibiting osteoclast formation *in vitro*. The results obtained indicate that TRs may be promising candidates for the effective treatment of osteoporosis caused by menopause in human females.

## Author Contributions

JS, XW, and YH conceived and designed the experiments. QL, ML, and XZ performed the experiments. YH, QL, JH, YW, and ML analyzed the data. JS and XW contribute dreagents, materials, and analysis tools. YH, QL, and XW wrote the manuscript. All authors read and approved the final manuscript.

## Conflict of Interest Statement

The authors declare that the research was conducted in the absence of any commercial or financial relationships that could be construed as a potential conflict of interest.

## Supplementary Material

FIGURE S1 The HPLC chromatogram of caffeine. **(B)** The standard curve of caffeine. **(C)** The HPLC chromatogram of BTE.

FIGURE S2 The HPLC chromatogram of TF **(A)**, TF-3′ **(C)**, TF-3,3′ **(E)**, BTE **(G)**. The standard curve of TF **(B)**, TF-3′ **(D)**, TF-3,3′ **(F)**.
