# Corrigendum: Effect of Black Tea Extract and Thearubigins on Osteoporosis in Rats and Osteoclast Formation *in vitro*

**DOI:** 10.3389/fphys.2020.00136

**Published:** 2020-02-25

**Authors:** Qingqing Liang, Ming Lv, Xiaojuan Zhang, Jun Hu, Ying Wu, Yewei Huang, Xuanjun Wang, Jun Sheng

**Affiliations:** ^1^Key Laboratory of Pu-er Tea Science, Ministry of Education, Yunnan Agricultural University, Kunming, China; ^2^Tea Research Center of Yunnan, Kunming, China; ^3^College of Food Science and Technology, Yunnan Agricultural University, Kunming, China; ^4^College of Science, Yunnan Agricultural University, Kunming, China; ^5^State Key Laboratory for Conservation and Utilization of Bio-Resources in Yunnan, Kunming, China

**Keywords:** osteoporosis, black tea extract, thearubigins, osteoclast, osteoclastogenesis

In the original article, there was a mistake in Figure 2 as published. Panels 2 and 3 (cortical bone tissue stained with H&E for Model and XLGB group, respectively) of Figure 2E in this paper are the same images as panels 2 and 3 of Figure 4D in Wang et al. (2018). Based on the 3R (Reduction, Replacement, and Refinement) principle of experimental animals, the authors simultaneously and systematically evaluated the pharmacological effects of Dendrobium officinale Orchid extract, black tea extract, and thearubigins in preventing osteoporosis using the same batch of ovariectomized (OVX) female rats as animal model of postmenopausal osteoporosis in the animal experiment study. They then collected the data and published two articles mentioned and this is how this error was introduced. The corrected Figure 2 appears below.

**Figure 2:**
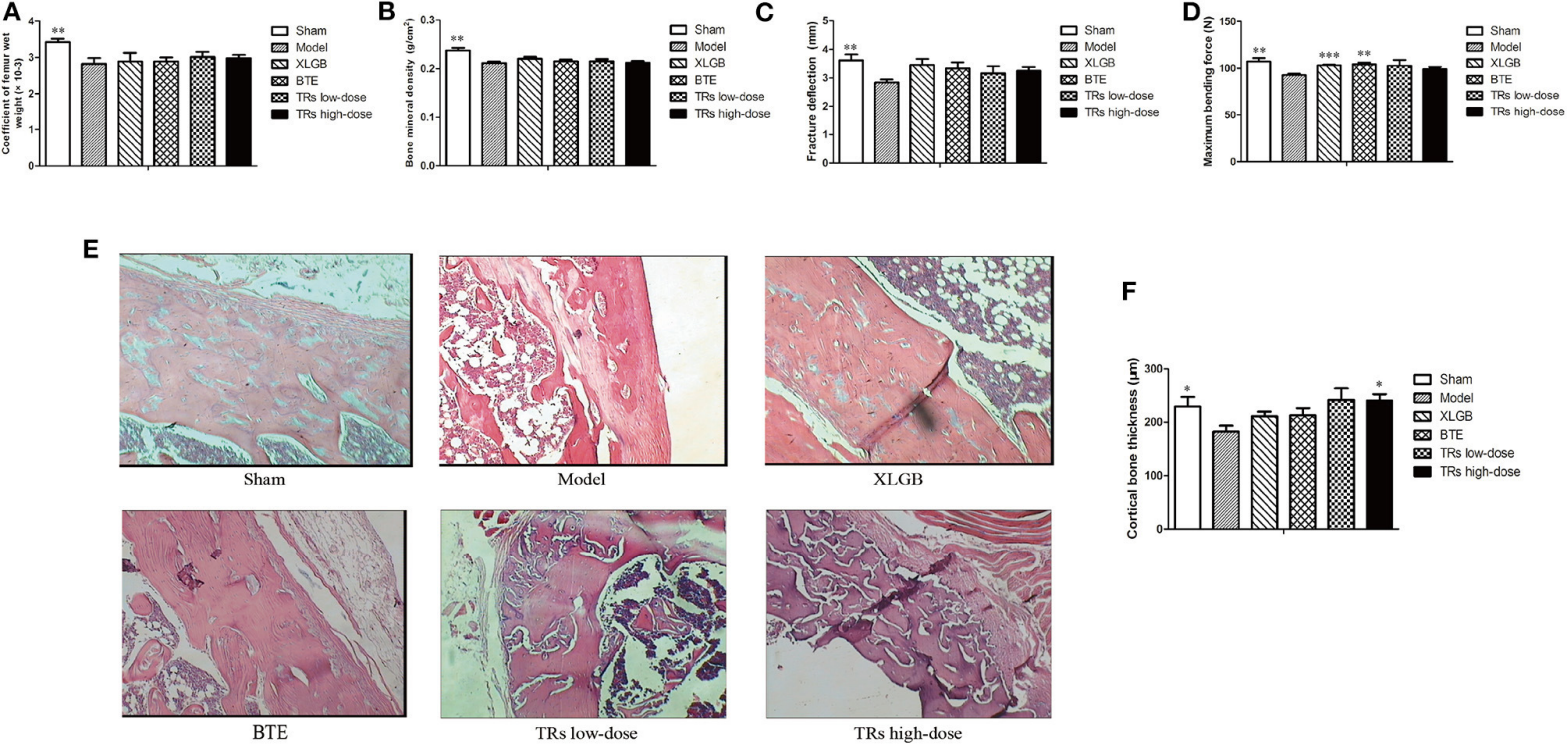
The effect of black tea extract (BTE) and thearubigins (TRs) on bone quality in ovariectomized (OVX) rats. **(A)** Femur wet weight coefficient, **(B)** bone mineral density, **(C)** femoral fracture deflection, **(D)** maximum bending force for each treatment group. **(E)** The cortical bone tissue was examined by hematoxylin and eosin (H&E) staining (magnification × 400) and **(F)** cortical bone thickness counts, statistical analysis. All data are presented as mean ± SEM (*n* = 10). “Independent Samples T-Test” and “One way ANOVA” were used for the comparison of sham vs model group and model vs treatment groups using pooled variance, respectively. *p < 0.05, **p < 0.01, and ***p < 0.001 vs model group.

The authors apologize for this error and state that this does not change the scientific conclusions of the article in any way. The original article has been updated.
