# Supplementary figures and images for: Effect of Black Tea Extract and Thearubigins on Osteoporosis in Rats and Osteoclast Formation in vitro
Source: Front Physiol. 2018 Sep 3;9:1225. doi: 10.3389/fphys.2018.01225 (PMC6129951; doi:10.3389/fphys.2018.01225)

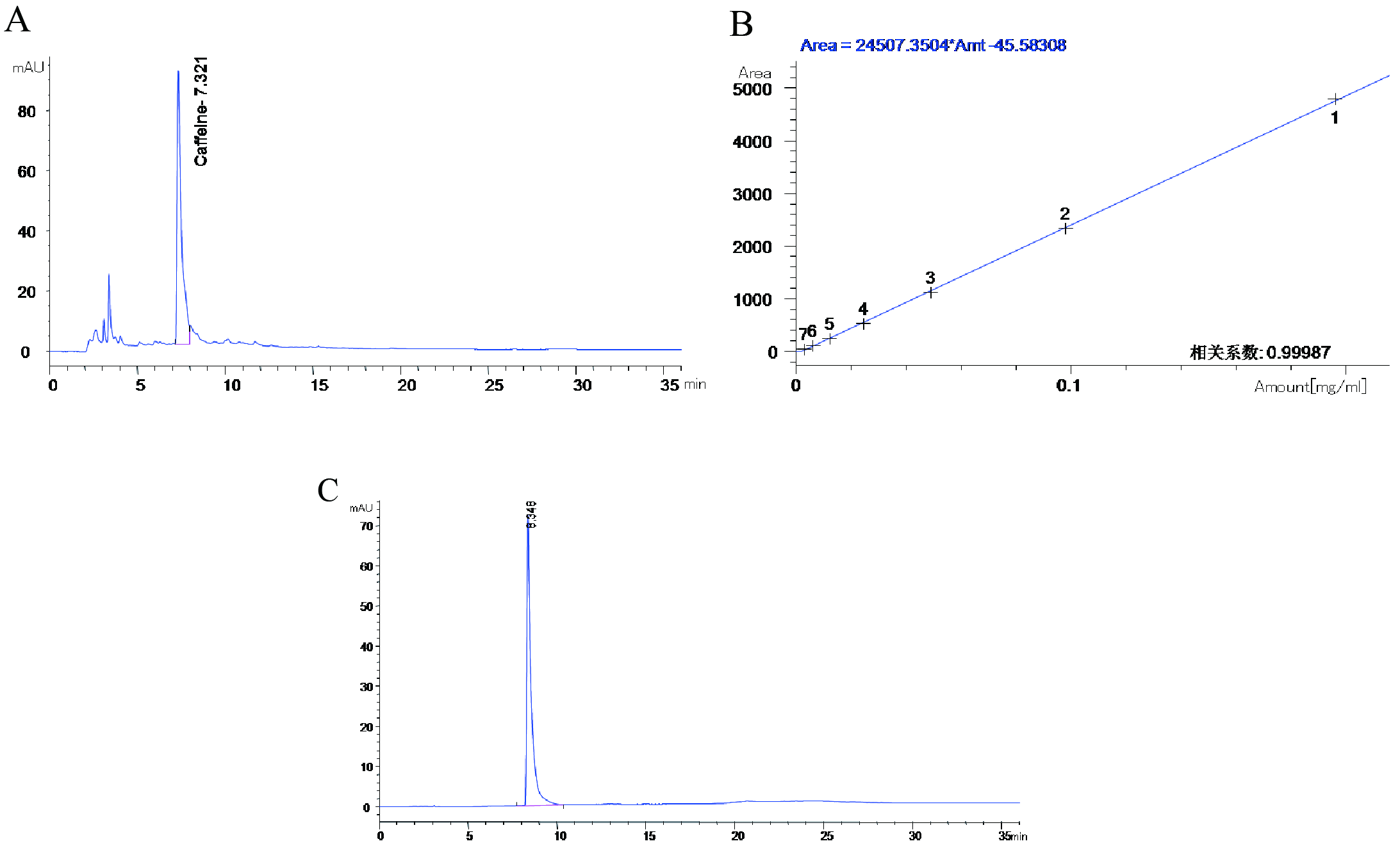

Supplement: FIGURE S1 — The HPLC chromatogram of caffeine. (B) The standard curve of caffeine. (C) The HPLC chromatogram of BTE. [file Image_1.TIF]

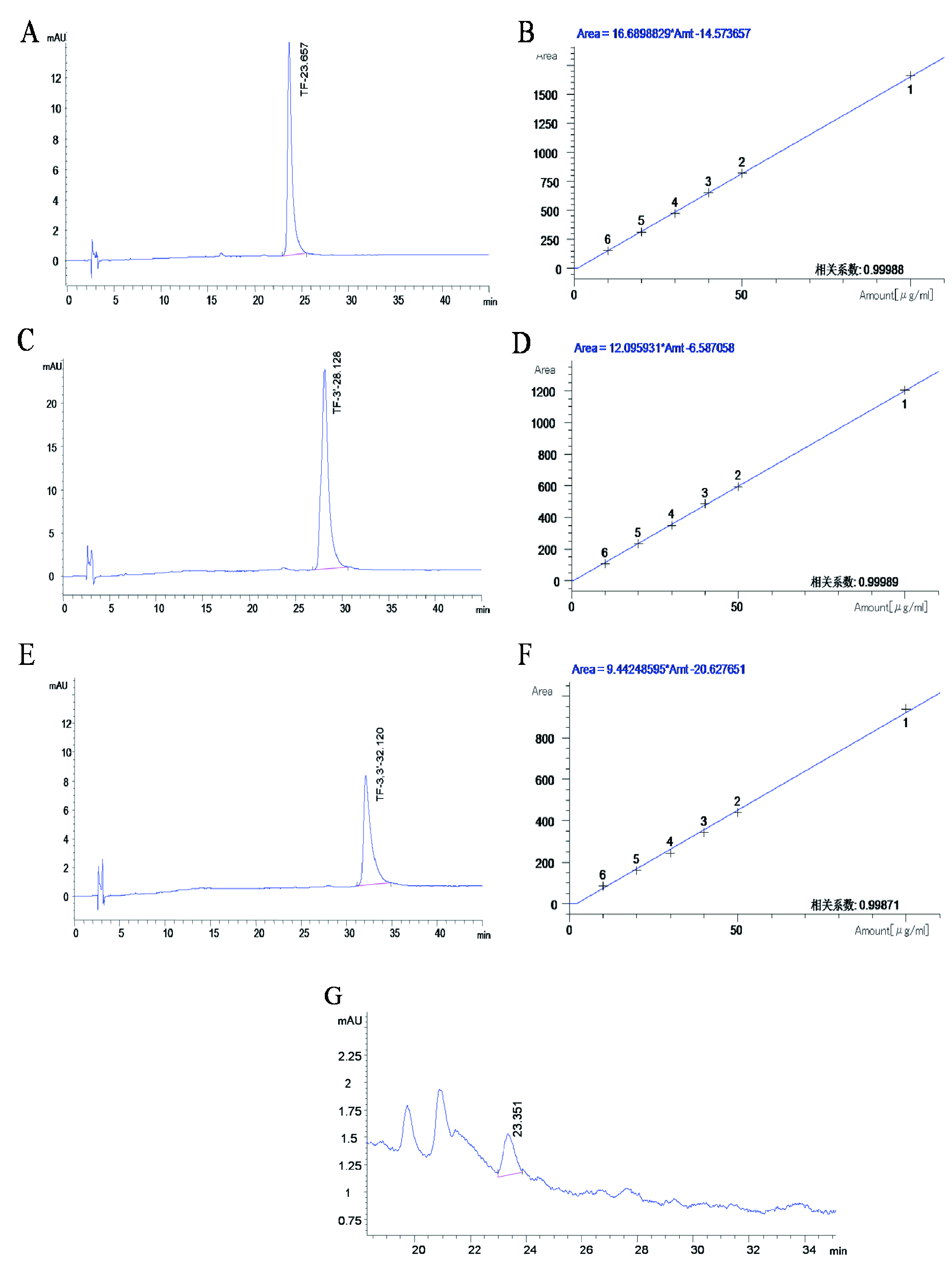

Supplement: FIGURE S2 — The HPLC chromatogram of TF (A), TF-3′ (C), TF-3,3′ (E), BTE (G). The standard curve of TF (B), TF-3′ (D), TF-3,3′ (F). [file Image_2.TIF]
