# Supplementary material for: Effect of Black Tea Extract and Thearubigins on Osteoporosis in Rats and Osteoclast Formation in vitro
Source: Front Physiol. 2018 Sep 3;9:1225. doi: 10.3389/fphys.2018.01225 (PMC6129951; doi:10.3389/fphys.2018.01225)
Supplement: Supplementary file 3 [file Data_Sheet_1.PDF]

Data analysis materials

The analysis of body weight data

The "Linear Mixed Models" was used to analyze the data of body weight. The model is as follows:

$$y_{ij} = \mu + \beta * week_i + \gamma * treat_{ij} + \varepsilon_{ij}, i = 1, \ldots, 12; j = 1, \ldots 13$$

y_{ij} represents the weight measurement of each individual at a time point.

$treat_{ij}$ represents fix effect and the group of each individual at a time point.

$week_i$ represents random effect and it reflected the differences between measurements at different times for the same individual, $week_i = (week_{i1}, \cdots, week_{i13})'$.

β, γ represents estimate of parameter, $\beta = (\beta_1, \ldots, \beta_{13})$, $\gamma = (\gamma_1, \gamma_2)$.

ε_{ij} represents the random error and follow normal distribution.

Note: *$week_{i13}$ represents the body weight of rats at the initial time and it as base line in our analysis.*

To analyze the effect of ovariectomy on the body weight of rats, we set the sham group to 'treat = 0' and model group to 'treat = 1'. In addition, to analyze the effect of treatments on the body weight of ovariectomized rats, we set the model group to 'treat = 0' and the specified treatment group to 'treat = 1'.

According to the model, the following results was obtained by SPSS (Table 1).

Table 1 The results of "Mixed Linear Models" analysis

**Comparison of Groups**	**The value range of** $\beta\,(\beta_1 \sim \beta_{13})$	**All of the Sig. of** $\beta\,(\beta_1 \sim \beta_{13})$	**The value and Sig. of** γ
Sham and Model	9.034~56.55	P<0.05	-27.15 (P=0.048)
Model and XLGB	13.74~73.65	P<0.05	-4.16 (P=0.812)
Model and BTE	10.60~77.25	P<0.05	5.46 (P=0.733)
Model and TRs-low	20.38~83.32	P<0.05	-14.696974 (P=0.453)
Model and TRs-high	15.01~67.55	P<0.05	11.74 (P=0.658)

Due to the value β was increasing over time and P<0.05 for all of the Sig. of β, the results showed that the body weight of the rats in each group was increasing over

time.
For the comparison of sham and model group, the body weight gain can be induced by ovariectomy ($\gamma = -27.15$, P=0.048).

However, there was no significant difference in body weight between model group and each treatment group (P>0.05). These results indicate that the OVX-induced body weight gain can not be down-regulated by the treatment with XLGB, BTE or TRs.

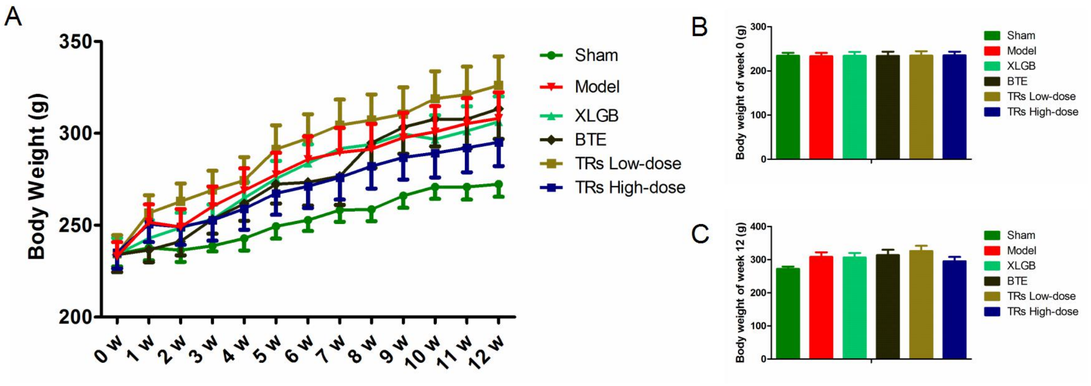

Figure 1 Effects of black tea extract (BTE) and thearubigins (TRs) on body weight (g) in OVX rats.

Moreover, the growth curve for each group and the average histogram for initial (week 0) and final body weight (week12) were also showed (Figure 1).
The results showed that the initial weights of the rats in each group were almost identical and the body weight of the rats in each group increased gradually during the 12 weeks. As expected, the body weight gain induced by ovariectomy was observed. The high-dose TRs had a tendency to down-regulate the OVX-induced body weight gain from the fourth week after ovariectomy.

The analysis of other indicators

For the comparison of sham and model group, SEM and “Independent Samples T-Test” was used to analyze the data. The results using pooled variance showed that there was significant difference in every indicator between Sham and model group (Table 2).

Table 2 The results of “Independent Samples T-Test” analysis

NO.	test items	Sig. (2-tailed)
1	Cortical bone thickness (μm)	0.044
2	BGP (μg/L)	<0.0001
3	ACP (μg/L)	<0.0001
4	Fracture deflection (mm)	0.005
5	Maximum bending force (N)	0.003
6	Coefficient of femur wet weight ($\times 10^{-3}$)	0.005
7	Bone mineral density (g/cm^2)	0.001

For the comparison of model and treatment groups, SEM and “One way ANOVA” was used to analyze the data. The results using pooled variance showed as follows (Table 3, see the revised manuscript for details).

Table 3 The results of “one way anova” analysis

NO.	test items	Sig. (2-tailed), compared with model group.			
		XLGB	BTE	TRs low-dose	TRs high-dose
1	Cortical bone thickness	0.436	0.614	0.241	0.02
2	BGP	0.724	0.452	0.977	0.982
3	ACP	0.597	0.785	0.954	0.002
4	Fracture deflection	0.171	0.481	0.948	0.242
5	Maximum bending force	<0.0001	0.001	0.804	0.313
6	Coefficient of femur wet weight	1	1	0.993	0.995
7	Bone mineral density	0.726	0.998	0.999	1
